# Benchmarking UK Lynch syndrome colonoscopy surveillance: quality metrics and diagnostic yield from the National Endoscopy Database

**DOI:** 10.1055/a-2939-3426

**Published:** 2026-08-31

**Authors:** Stephanie X. W. Poo, Thomas Lee, Amanda J. Cross, Kevin J. Monahan

**Affiliations:** 1 Centre for Familial Intestinal Cancer 105692 St Mark's Hospital and Academic InstituteLondonUnited Kingdom of Great Britain and Northern Ireland; 2 Department of Surgery and Cancer 4615 Imperial College LondonLondonUnited Kingdom of Great Britain and Northern Ireland; 3 Department of Gastroenterology 6072 Northumbria Healthcare NHS Foundation TrustNorth ShieldsUnited Kingdom of Great Britain and Northern Ireland

## Abstract

**Background:**

Colonoscopy quality and diagnostic outcomes in Lynch syndrome have historically been heterogeneous. Following the introduction of the Lynch syndrome Bowel Cancer Screening Programme (BCSP), we assessed colonoscopy quality and diagnostic yield in Lynch syndrome using the National Endoscopy Database (NED), comparing outcomes between BCSP and non-BCSP providers.

**Methods:**

We performed a cross-sectional analysis of UK Lynch syndrome colonoscopies from NED between June 2024 and June 2025. Colonoscopy quality indicators and endoscopic detection rates for polyp (PDR) and advanced neoplasia (eANDR; polyps ≥10 mm or cancer; histopathology linkage was unavailable) were calculated and compared between BCSP and non-BCSP providers. Factors associated with neoplasia detection were identified using logistic regression.

**Results:**

Among 3196 colonoscopies, most were performed in England (90.2%), with 34.1% undertaken within BCSP. Overall colonoscopy quality was high: 97.6% were complete, 89.0% had adequate bowel preparation, and 96.3% had adequate tolerance. BCSP procedures demonstrated higher rates of completion (98.5% vs. 97.1%; P = 0.02) and adequate bowel preparation (92.7% vs. 86.7%; P < 0.001) than non-BCSP colonoscopies. PDR and eANDR were 46.8% and 4.7%, respectively, and were higher in BCSP than non-BCSP procedures (PDR 53.7% vs. 43.3%; eANDR 5.7% vs. 3.9%). Higher polyp detection was associated with increasing age (odds ratio [OR] 1.03, 95%CI 1.03–1.04), male gender (OR 1.48, 95%CI 1.26–1.73), BCSP status (OR 1.50, 95%CI 1.28–1.77), adequate bowel preparation (OR 1.81, 95%CI 1.40–2.34), and England nation (OR 1.99, 95%CI 1.22–3.36).

**Conclusions:**

Our NED evaluation demonstrated high colonoscopy performance in Lynch syndrome, with superior quality and endoscopic yield in BCSP procedures, supporting national integration of surveillance to establish Lynch syndrome-specific quality benchmarks.

## Abstract (de)

**Graphical abstract:**

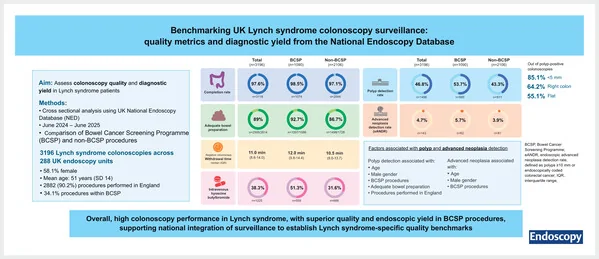

## Introduction

Lynch syndrome is a hereditary cancer predisposition syndrome caused by pathogenic variants in one of five DNA mismatch repair (MMR) genes, resulting in a markedly increased risk of colorectal cancer (CRC) and other extracolonic malignancies 1 2 . Colonoscopic surveillance is the cornerstone of CRC prevention and early detection in Lynch syndrome, and has been shown to reduce CRC incidence by >50% and mortality by 65% 3 . UK guidelines recommend biennial colonoscopies from 25 years for *MLH1* , *MSH2* , and *EPCAM* carriers, and from 35 years for *MSH6* and *PMS2* variants 4 . As the effectiveness of surveillance relies on timely and high-quality examinations, ensuring consistently high colonoscopy standards is critical for this high-risk population.

In routine practice, key performance indicators have been defined by international societies such as the British Society of Gastroenterology (BSG) and European Society of Gastrointestinal Endoscopy (ESGE), including cecal intubation rate, bowel preparation quality, withdrawal time, patient comfort, and adenoma detection rate (ADR) 5 6 7 . These benchmarks have driven measurable improvements across routine endoscopy services, although meaningful variation in quality persists 8 . To support evaluation of these key performance indicators in the UK, the National Endoscopy Database (NED) was developed to prospectively capture standardized procedure-level data from endoscopy reporting systems, thus enabling routine audit and monitoring of endoscopy performance 9 .

Since 2013, NED has spearheaded several key UK-wide analyses, including evaluations of endoscopy workforce capacity and service demand, diagnostic yield of symptomatic upper and lower gastrointestinal endoscopies, quality assessments, and interventional work using live feedback to improve colonoscopy performance 8 10 11 12 13 . These large-scale outputs demonstrate the utility of NED in systematically evaluating endoscopy practices in the UK without reliance on manual data collection. However, NED remains limited by lack of histopathology linkage, patient-level data, and longitudinal data, restricting analyses to cross-sectional and surrogate performance measures only. Thus, polyp detection rate (PDR) is used as an alternative to ADR as it correlates strongly with adenoma detection and has comparable associations with post-colonoscopy CRC outcomes 14 15 16 17 . The ability of NED to capture near-complete procedure-level data on a national scale provides a unique opportunity to evaluate and benchmark colonoscopy quality in all populations, including Lynch syndrome.

Given the increased and often early-onset CRC risks in Lynch syndrome, together with accelerated neoplastic progression and predilection for flat right-sided lesions, meticulous mucosal evaluations and effective polypectomy techniques are even more essential 18 19 . Furthermore, because patients with Lynch syndrome need to undergo repeated colonoscopies throughout their lifetime, patient comfort and tolerability are key to ensuring long-term adherence to surveillance. Nonetheless, existing key performance indicators were developed primarily for average-risk populations and may not sufficiently capture the quality standards required for Lynch syndrome surveillance. Historical Lynch syndrome surveillance outcomes have been heterogeneous, with most data derived from specialist or academic centers, and limited by inconsistent reporting of key performance indicators. To date, no Lynch syndrome-specific quality benchmarks have been established and population-level data on quality-controlled surveillance remain poorly characterized.

Incorporation of Lynch syndrome colonoscopic surveillance into the English National Health Service (NHS) Bowel Cancer Screening Programme (BCSP) in July 2023 offers a national framework to embed systematic quality assurance within Lynch syndrome surveillance pathways 20 21 . Details of the BCSP accreditation processes have been described previously 21 22 . In brief, the NHS BCSP operates within a structured, quality-assured environment whereby surveillance colonoscopies are performed by accredited endoscopists, with specialist screening practitioner support, enhanced governance processes, and longer appointment times 21 22 23 . However, the impact of Lynch syndrome BCSP on surveillance quality, and how BCSP procedures compare with non-BCSP colonoscopies nationally, have not been evaluated. Using NED, we aimed to characterize Lynch syndrome colonoscopy quality and endoscopic yield across the UK and compare performance between BCSP- and non-BCSP procedures to inform future Lynch syndrome-specific benchmarks and quality standards.

## Methods

### Study design and data acquisition

NED was established under the governance of the Joint Advisory Group on Gastrointestinal Endoscopy, and provides a national platform for automated capture of endoscopy data with its second iteration (NEDi2) expanding data acquisition across other endoscopic procedures (full details published elsewhere) 9 .

A cross-sectional analysis was undertaken, extracting colonoscopy records uploaded in NEDi2 for adults (≥18 years) with Lynch syndrome between 1 June 2024 and 30 June 2025. This study duration was chosen to reduce duplications arising from repeated surveillance procedures in the absence of unique patient identifiers. Lynch syndrome cases were identified through the “High cancer risk surveillance” indication field, and “Lynch syndrome” sub-indication, and by searching the free-text fields in each Indication and Diagnosis column. Procedures labeled as “Lynch-like syndrome,” “LS excluded,” “suspected/possible Lynch syndrome,” or “family history of Lynch syndrome,” were excluded.

### Outcomes

Colonoscopy quality indicators were assessed according to BSG/ESGE standards (see **Table 1s** in the online-only Supplementary material) 5 7 . Both overall withdrawal time and withdrawal time of procedures without pathology (negative colonoscopy withdrawal time) were recorded. Information on contributing endoscopists, including professional role and procedure volume, over the study period was extracted.

Endoscopic yield including PDR and endoscopically defined advanced neoplasia (eANDR; defined as polyps ≥10 mm or endoscopically coded CRC, or both) detection rate were evaluated. Mean number of polyps per all procedures and per positive procedure (colonoscopies with ≥1 polyp identified) were calculated, and site-level and regional distribution of PDR were analyzed. Moreover, polyp characteristics including size, morphology according to the Paris classification, and anatomic location were analyzed. As NED does not capture histopathology outcomes, it was not possible to calculate the ADR; thus, PDR was used as a surrogate marker, and an ADR-to-PDR conversion factor of 0.688 was applied to estimate the ADR 16 .

The CRC detection rate was not reported because of the preponderance of early-stage CRCs – including T1 cancers – in Lynch syndrome, which may be recorded endoscopically as large polyps rather than overt cancers 24 25 . Published Lynch syndrome surveillance cohorts have consistently demonstrated a high burden of early-stage CRC, with 44%–58% of cancers diagnosed at stage 1 across MMR variants, and a predominance of T1 cancers 24 25 26 27 . Without histopathology, reliable distinction between advanced adenomas, malignant polyps, and early invasive CRC is not possible, and reporting CRC detection rate using endoscopic coding could result in underestimation of cancer burden; therefore, eANDR was used as a composite end point.

Quality parameters and diagnostic outcomes were compared between BCSP and non-BCSP procedures, with BCSP status defined according to procedure-level coding within NED rather than center designation. Gender-stratified analyses included only participants recorded as male or female, while procedures with gender coded as ‘Other’ were excluded due to small numbers. Diagnostic outcomes were also stratified by presumed history of proximal colorectal resections, inferred from documented extent of examination to the ileo-colonic anastomosis or neo-terminal ileum.

### Statistical analysis

Descriptive statistics were used to summarize demographic, procedural, and outcome variables. We performed chi-squared analyses to test for associations between categorical variables and employed Fisher’s exact tests where expected cell counts were <10. Continuous variables with normal distributions were summarized as mean (SD) and compared using two-sample *t* test, while non-normally distributed data were summarized as median (interquartile range [IQR]) and compared using Wilcoxon rank-sum test. Multivariable logistic regression analysis was performed to identify factors associated with increased PDR and eANDR. Variables included in the model were selected based on clinical relevance, and results were presented as odds ratios (ORs) with 95%CI. A *P* value of <0.05 was considered statistically significant. No formal adjustment for multiple testing was performed. All statistical analyses were performed using RStudio version 2024.12.0+467 (Posit Software, PBC, Boston, Massachusetts, USA).

## Results

Of the 525 UK endoscopy units, 499 (95.0%) contributed data to NED, and 288 centers (54.9%) and 978 endoscopists completed 3196 colonoscopies on patients with Lynch syndrome. Baseline demographics, procedure indications, quality indicators, and procedure-level endoscopists’ data are summarized in Table 1 , and characteristics of participating endoscopists, including procedure volume, are presented in **Table 2s** . The mean age was 51.4 (SD 13.6) years, and 1856 procedures (58.1%) were undertaken in female patients. Most colonoscopies (90.2%) were performed in England, followed by Wales (4.9%), Scotland (4.0%), and Northern Ireland (0.9%). The majority were undertaken for surveillance (n = 2975, 93.1%), while 221 (6.9%) were performed for symptomatic indications: positive fecal immunochemical test or fecal occult blood test (n = 133, 60.2%), rectal bleeding (n = 41, 18.6%), changes in bowel habit (n = 36, 16.3%), abdominal pain (n = 29, 13.1%), anemia (n = 12, 5.4%), weight loss (n = 10, 4.5%), and abnormal imaging investigations (n = 2, 0.9%).

**Table:** **Table 1** Colonoscopy demographics and quality indicators stratified by Bowel Cancer Screening Programme (BCSP) and non-BCSP procedures.

Variable	Total, n (%)	BCSP, n (%)	Non-BCSP, n (%)	*P* value
Patients	3196	1090 (34.1)	2106 (65.9)	
Gender ^1^				0.24 ^1^
Male	1305 (40.8)	455 (41.7)	850 (40.4)	
Female	1856 (58.1)	609 (55.9)	1247 (59.2)	
Other	35 (1.1)	26 (2.4)	9 (0.4)	
Age, years				0.06
Mean (SD)	51.4 (13.6)	50.8 (12.7)	51.7 (13.9)	
18–24	25 (0.8)	0 (0.0)	25 (1.2)	
25–34	358 (11.2)	112 (10.3)	246 (11.7)	
35–44	681 (21.3)	268 (24.6)	413 (19.6)	
45–54	741 (23.2)	264 (24.2)	477 (22.7)	
55–64	793 (24.8)	265 (24.3)	528 (25.1)	
65–74	486 (15.2)	170 (15.6)	316 (15.0)	
75–84	108 (3.4)	11 (1.0)	97 (4.6)	
≥85	4 (0.1)	0 (0.0)	4 (0.2)	
UK region				<0.001
Northern Ireland	28 (0.9)	0 (0.0)	28 (1.3)	
Scotland	128 (4.0)	1 (0.1)	127 (6.0)	
Wales	158 (4.9)	7 (0.6)	151 (7.2)	
England	2882 (90.2)	1082 (99.3)	1800 (85.5)	
East Midlands	313 (9.8)	132 (12.1)	181 (8.6)	
East of England	426 (13.3)	123 (11.3)	303 (14.4)	
London	453 (14.2)	139 (12.8)	314 (14.9)	
North East England	187 (5.9)	73 (6.7)	114 (5.4)	
North West England	301 (9.4)	96 (8.8)	205 (9.7)	
South East England	336 (10.5)	132 (12.1)	204 (9.7)	
South West England	409 (12.8)	165 (15.1)	244 (11.6)	
West Midlands	263 (8.2)	146 (13.4)	117 (5.6)	
Yorkshire and Humber	194 (6.1)	76 (7.0)	118 (5.6)	
Indication				0.07
Surveillance	2975 (93.1)	1002 (91.9)	1973 (93.7)	
Symptomatic	221 (6.9)	88 (8.1)	133 (6.3)	
Positive FIT/FOBT	133 (60.2)	85 (96.6)	48 (36.1)	
Rectal bleeding	41 (18.6)	1 (1.1)	40 (30.1)	
Bowel habit changes	36 (16.3)	1 (1.1)	35 (26.3)	
Abdominal pain	29 (13.1)	0 (0.0)	29 (21.8)	
Anemia	12 (5.4)	1 (1.1)	11 (8.3)	
Weight loss	10 (4.5)	0 (0.0)	10 (7.5)	
Abnormal imaging	2 (0.9)	0 (0.0)	2 (1.5)	
Endoscopists				<0.001
Consultant	1780 (55.7)	631 (57.9)	1149 (54.6)	
Clinical Endoscopist	641 (20.1)	233 (21.4)	408 (19.4)	
Non-consultant/Other staff	118 (3.7)	31 (2.8)	87 (4.1)	
Trainee	64 (2.0)	6 (0.6)	58 (2.8)	
Unknown	593 (18.6)	189 (17.3)	404 (19.2)	
Data are n (%), unless otherwise specified. BCSP, Bowel Cancer Screening Programme; FIT, fecal immunochemical test; FOBT, fecal occult blood test. ^1^ Patient-identified gender analyses included male and female participants only; “Other” genders were excluded.				

BCSP colonoscopies accounted for 1090 procedures (34.1%), while 2106 (65.9%) were performed outside BCSP ( Table 1 ). Median procedure volume of endoscopists performing ≥1 BCSP colonoscopy was higher than those undertaking no BCSP procedures (415 [IQR 284–614] vs. 315 [IQR 183–526]; *P* < 0.001) ( **Table 2s** ). There were no significant differences in patient gender, age distribution, and procedure indication between groups, although a broader age range was observed in the non-BCSP cohort. Almost all BCSP procedures were undertaken in England (1082/1090, 99.3%), compared with 1800/2106 (85.5%) non-BCSP colonoscopies. Fig. 1 shows the regional distribution of UK Lynch syndrome colonoscopies, stratified by BCSP and non-BCSP groups.

**Fig. 1:**
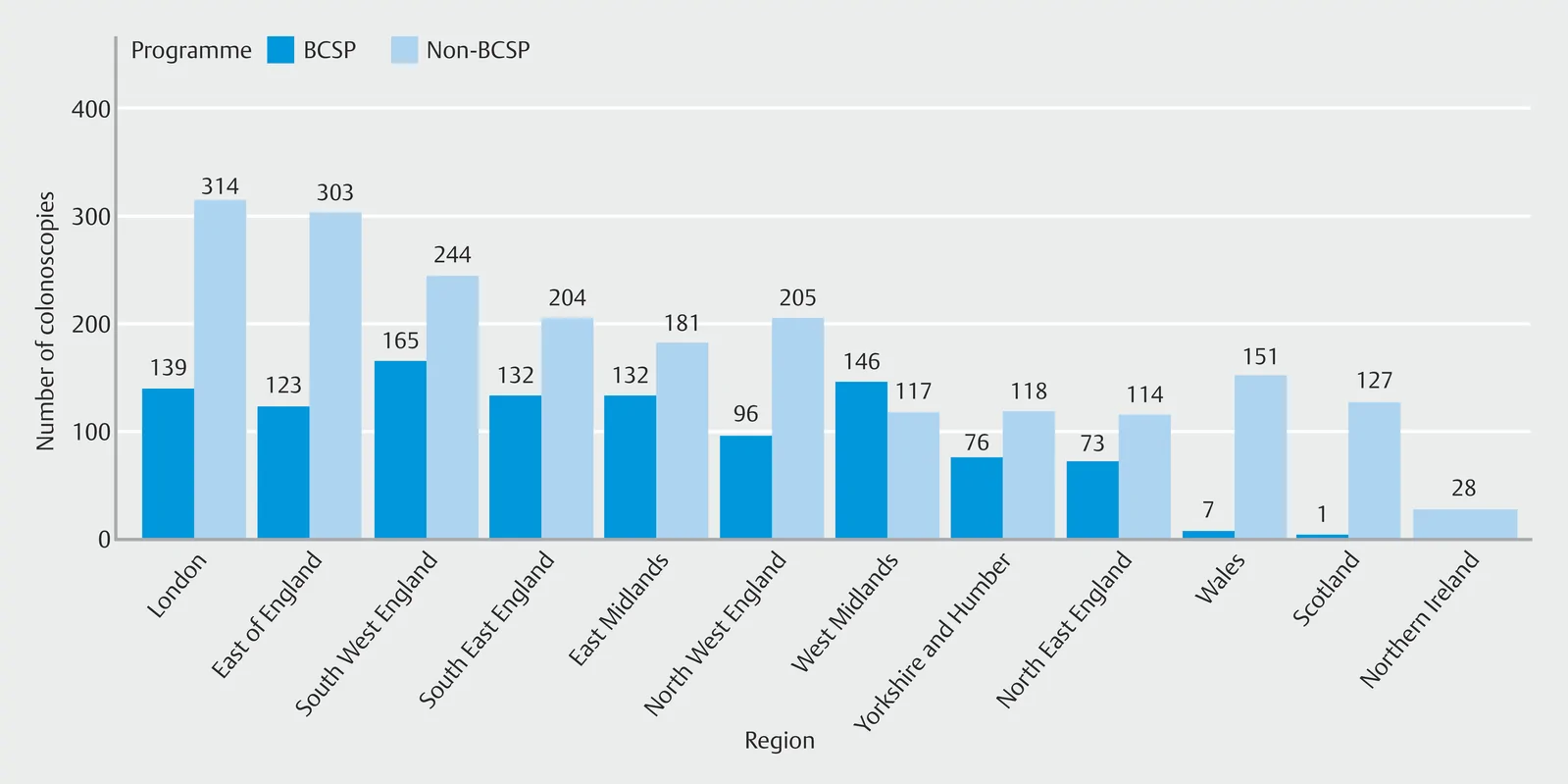
Regional distribution of colonoscopies performed in Lynch syndrome patients, stratified by BCSP and non-BCSP groups. BCSP, Bowel Cancer Screening Programme.

### Colonoscopy quality indicators

Table 2 demonstrates quality indicators for colonoscopies performed on patients with Lynch syndrome. Complete procedures were achieved in 3118/3196 colonoscopies (97.6%). Of these, the cecum or terminal ileum was reached in 2731 procedures (87.6%), and ileo-colon anastomosis or neo-terminal ileum intubation was attained in 387 cases (12.4%). Adequate bowel preparation was documented in 2505/2814 procedures (89.0%; mean Boston Bowel Preparation Scale score 7.4 [SD 2.0]). Median withdrawal time was 13.1 minutes (IQR 10.0–18.0) overall and 11.0 minutes (IQR 8.6–14.0) for negative colonoscopies. Among negative colonoscopies, the withdrawal time was <10 minutes in 636 procedures (44.2%), ≥10 minutes in 802 (55.8%), and >14 minutes in 306 (21.3%).

**Table:** **Table 2** Colonoscopy quality parameters for patients with Lynch syndrome undergoing colonoscopy stratified by Bowel Cancer Screening Programme (BCSP) and non-BCSP procedures.

	Total, N = 3196	BCSP, N = 1090	Non-BCSP, N = 2106	*P* value
Complete procedures	3118 (97.6)	1074 (98.5)	2044 (97.1)	0.02
Cecum	1416 (44.3)	486 (44.6)	930 (44.2)	
Terminal ileum	1315 (41.2)	468 (42.9)	847 (40.2)	
Neo-terminal ileum	257 (8.0)	77 (7.1)	180 (8.5)	
Ileo-colon anastomosis	130 (4.1)	43 (3.9)	87 (4.1)	
Adequate bowel preparation	2505/2814 (89.0)	1007/1086 (92.7)	1498/1728 (86.7)	<0.001
BBPS score, mean (SD)	7.4 (2.0)	7.5 (1.8)	7.3 (2.0)	
Withdrawal time, minutes				
Overall, median (IQR)	13.1 (10.0–18.0)	13.8 (10.3–18.7)	11.6 (8.0–16.3)	<0.001
Negative colonoscopy				<0.001
Median (IQR)	11.0 (8.6–14.0)	12.0 (9.8–14.4)	10.5 (8.0–13.7)	
<6	250/1438 (17.4)	73/502 (14.5)	177/936 (18.9)	
6–<10	386/1438 (26.8)	100/502 (19.9)	286/936 (30.6)	
10–14	496/1438 (34.5)	205/502 (40.8)	291/936 (31.1)	
>14	306/1438 (21.3)	124/502 (24.7)	182/936 (19.4)	
Adequate tolerance	2674/2777 (96.3)	1048/1085 (96.6)	1626/1692 (96.1)	0.57
Sedation/analgesic use				
Unsedated	882 (27.6)	283 (26.0)	599 (28.4)	0.15
Nitrous oxide	1022 (32.0)	369 (33.9)	653 (31.0)	0.11
Conscious sedation	2276 (71.2)	804 (73.8)	1472 (69.9)	0.03
Fentanyl, mean (SD), µg	63.4 (21.6)	64.4 (21.2)	62.9 (21.8)	0.13
Midazolam, mean (SD). mg	2.2 (0.7)	2.2 (0.7)	2.3 (0.7)	0.006
Pethidine, mean (SD), mg	44.1 (13.3)	42.5 (11.6)	46.8 (15.5)	0.19
General anesthetic	69 (2.2)	8 (0.7)	61 (2.9)	<0.001
Hyoscine butylbromide	1225 (38.3)	559 (51.3)	666 (31.6)	<0.001
Data are n (%) or n/N (%), unless otherwise specified. BCSP, Bowel Cancer Screening Programme; BBPS, Boston Bowel Preparation Scale; IQR, interquartile range.				

Adequate tolerance was reported in 2674/2777 colonoscopies (96.3%). In this cohort, 882 procedures (27.6%) were unsedated, while 1022 (32.0%) used nitrous oxide, 2276 (71.2%) used conscious sedation, and 69 (2.2%) were performed under general anesthesia. Fentanyl was administered in 2093 (65.5%), midazolam in 2224 (69.6%), and pethidine in 80 (2.5%) cases, while concomitant fentanyl and midazolam were used in 2032 procedures (63.6%). Corresponding mean (SD) doses were 63.4 (21.6) µg for fentanyl, 2.2 (0.7) mg for midazolam, and 44.1 (13.3) mg for pethidine. Intraprocedural hyoscine butylbromide was used in 1225 colonoscopies (38.3%).

BCSP procedures demonstrated superior quality metrics compared with non-BCSP colonoscopies ( Table 2 ), including higher completion rates (98.5% vs. 97.1%, chi-squared = 5.967, *P* = 0.02), and greater proportion with adequate bowel preparation (92.7% vs. 86.7%; chi-squared = 24.241, *P* < 0.001). Median overall withdrawal time was longer in BCSP procedures (13.8 vs. 11.6 minutes; Wilcoxon rank-sum test *P* < 0.001), as was the median negative colonoscopy withdrawal time (12.0 vs. 10.5 minutes; Wilcoxon rank-sum test *P* < 0.001). Among negative colonoscopies, 329 BCSP procedures (65.5%) achieved a withdrawal time of ≥10 minutes compared with 473 non-BCSP colonoscopies (50.5%; chi-squared = 29.433, *P* < 0.001). There were no significant differences in procedure tolerance and sedation use between groups; however, intravenous hyoscine butylbromide was more frequently used among BCSP colonoscopies (51.3% vs. 31.6%; chi-squared = 117.0, *P* < 0.001).

### Colonoscopic yield

PDR was 46.8% (95%CI 45.1–48.5) and eANDR was 4.7% (95%CI 3.8–5.2) ( Table 3 ). There was a stepwise increase in the mean PDR with increasing colonoscopy volume, rising from 40% in low-volume centers (0–25 procedures) to nearly 60% in sites performing >100 colonoscopies, with reduced variability observed at higher volumes, although the number of high-volume centers was small ( Fig. 2 **a** ). At a regional level, there was significant variation in PDR (chi-squared = 39.999, *P* < 0.001), with English regions accounting for overall higher colonoscopy volumes and higher PDR compared with devolved nations ( Fig. 2 **b** ).

**Table:** **Table 3** Colonoscopic yield and polyp characteristics.

Variable	Total, n (%)	BCSP, n (%)	Non-BCSP, n (%)	*P* value
Colonoscopies	3196	1090 (34.1)	2106 (65.9)	
Diagnosis				<0.001
Normal	1260 (39.4)	371 (34.0)	889 (42.2)	
Polyp (PDR)	1496 (46.8)	585 (53.7)	911 (43.3)	
Advanced neoplasia (eANDR) ^1^	143 (4.5)	62 (5.7)	81 (3.9)	
Other benign conditions ^2^	420 (13.1)	128 (11.7)	292 (13.9)	
Benign diagnoses ^2^				
Diverticulosis	492 (15.4)	185 (17.0)	307 (14.6)	
Hemorrhoids	255 (8.0)	90 (8.3)	165 (7.8)	
Post-operative appearance	115 (3.6)	37 (3.4)	78 (3.7)	
Inflammatory conditions	32 (1.0)	12 (1.1)	20 (0.9)	
Anal tag	16 (0.5)	7 (0.6)	9 (0.4)	
Angioectasia	14 (0.4)	5 (0.5)	9 (0.4)	
Ulceration not otherwise specified	12 (0.4)	4 (0.4)	8 (0.4)	
Lipoma	7 (0.2)	4 (0.4)	3 (0.1)	
Polyp characteristics				
No. polyps per colonoscopy, mean (SD)				
All colonoscopies	1.08 (1.83)	1.25 (1.78)	0.99 (1.85)	<0.001
Positive colonoscopies	2.29 (2.09)	2.31 (1.85)	2.28 (2.22)	0.79
Size, mm				
<5	1177/1383 (85.1)	470/556 (84.5)	707/827 (85.5)	
5–9	353/1383 (25.5)	179/556 (32.2)	174/827 (21.0)	
≥10	154/1383 (11.1)	62/556 (11.2)	92/827 (11.1)	>0.99
Morphology				
Sessile	698/1233 (56.6)	333/556 (59.9)	365/677 (53.9)	
Flat	679/1233 (55.1)	308/556 (55.4)	371/677 (54.8)	<0.001
Paris IIa	648/1233 (52.6)	303/556 (54.5)	345/677 (51.0)	<0.001
Paris IIb	39/1233 (3.2)	14/556 (2.5)	25/677 (3.7)	0.62
Paris IIa+IIc	2/1233 (0.2)	0 (0.0)	2/677 (0.3)	0.52
LST-G	6/1233 (0.5)	0 (0.0)	6/677 (0.9)	0.09
LST-NG	2/1233 (0.2)	0 (0.0)	2/677 (0.3)	0.52
Pedunculated	24/1233 (2.0)	11/556 (2.0)	13/677 (1.9)	
Location				
Right colon	880/1370 (64.2)	383/555 (69.0)	497/815 (61.0)	0.001
Left colon	670/1370 (48.9)	279/555 (50.3)	391/815 (48.0)	
Rectum	276/1370 (20.2)	112/555 (20.2)	164/815 (20.1)	
Right-sided flat polyps	431/1222 (35.3)	206/555 (37.1)	225/667 (33.7)	<0.001
Flat polyps ≥10 mm	65/1233 (5.3)	20/556 (3.6)	45/677 (6.7)	0.15
Data are n (%) or n/N (%), unless otherwise specified. Denominators represent the number of polyp-positive procedures with available data for the characteristic of interest. Size, morphology, and location categories represent procedures containing ≥1 polyp of the specified characteristic. As individual procedures may contain >1 polyp with a given characteristic, categories are not mutually exclusive and cumulative percentages may not sum up to 100%. Among procedures with ≥1 flat lesion, subtype categories according to Paris classification were analyzed at lesion level, and a single procedure may contribute to >1 Paris subtype category. LST, laterally spreading tumor; eANDR, endoscopic advanced neoplasia detection rate; G, granular type; NG, nongranular type; PDR, polyp detection rate. ^1^ eANDR is endoscopically defined as polyps ≥10 mm or endoscopically coded colorectal cancer. ^2^ Each procedure can have more than one diagnosis.				

**Fig. 2:**
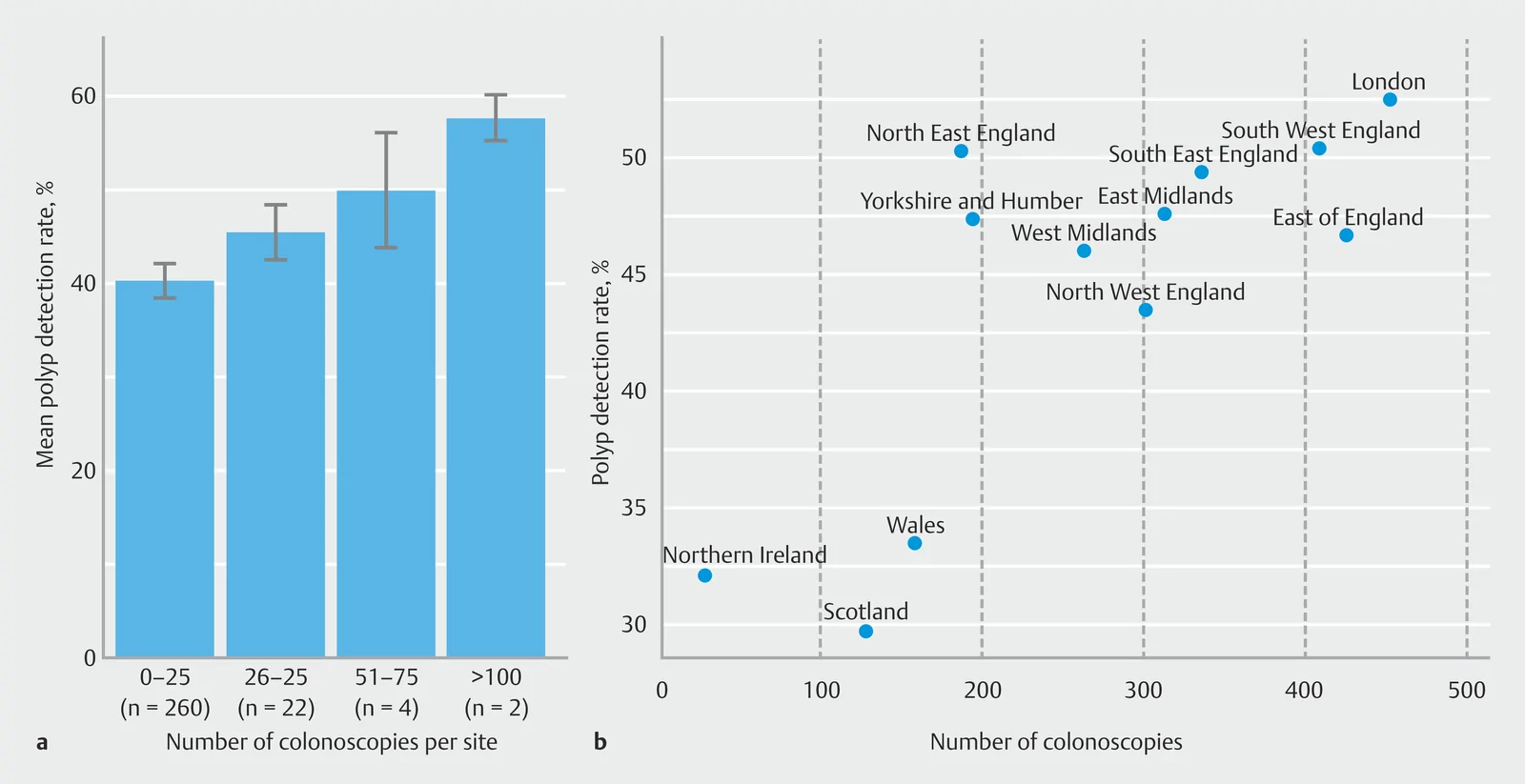
Polyp detection rates: **a** stratified by endoscopy volume; **b** stratified by region.

A total of 1260 procedures (39.4%) were normal while 420 colonoscopies (13.1%) reported other benign conditions, although diagnostic categories were not mutually exclusive in the latter ( Table 3 ). Of benign findings, diverticulosis was most commonly reported (n = 492, 15.4%) ( Table 3 ). PDR and eANDR in patients with previous proximal colorectal resections were 47.5% and 4.4%, respectively, with no significant differences in diagnostic outcomes between those who had prior resections and those without.

Mean number of polyps was 1.08 (SD 1.83) per procedure and 2.29 (SD 2.09) per procedure among positive colonoscopies ( Table 3 ). Of polyp-positive colonoscopies, 1177/1383 (85.1%) identified polyps <5 mm, while 353/1383 (25.5%) had polyps 5–9 mm, and 154/1383 (11.1%) had polyps ≥10 mm. Sessile polyps occurred in 56.6% of colonoscopies with polyps, and 55.1% reported flat polyps. Among flat polyps, morphology was Paris IIa in 648/1233 procedures (52.6%), whereas laterally spreading tumors (8/1233, 0.6%) and Paris IIa+IIc lesions (2/1233, 0.2%) were uncommon. Polyps were frequently right sided (880/1370, 64.2%), with 670/1370 (48.9%) and 276/1370 (20.2%) originating from the left colon and rectum, respectively. Right-sided flat lesions were present in 431/1222 procedures (35.3%), while flat polyps ≥10 mm were identified in 65/1233 colonoscopies (5.3%).

PDR and eANDR were significantly higher in BCSP colonoscopies compared with colonoscopies performed by non-BCSP providers (PDR 53.7% [95%CI 50.7–56.6] vs. 43.3% [95%CI 41.2–45.4]; chi-squared = 30.859, *P* < 0.001; eANDR 5.7% [95%CI 4.5–7.2] vs. 3.9% [95%CI 3.1–4.8]; chi-squared = 5.278, *P* = 0.02). BCSP procedures had higher detection rates of flat and right-sided polyps compared with non-BCSP colonoscopies: flat PDR 28.3% vs. 17.6% (chi-squared = 14.346, *P* < 0.001), right-sided PDR 35.1% vs. 23.6% (chi-squared = 10.719, *P* = 0.001), and right-sided flat PDR 18.9% vs.10.7% (chi-squared = 14.562, *P* < 0.001).

Multivariable logistic regression showed higher polyp detection was associated with increasing age (OR 1.03, 95%CI 1.03–1.04), male gender (OR 1.48, 95%CI 1.26–1.73), BCSP status (OR 1.50, 95%CI 1.28–1.77), adequate bowel preparation (OR 1.81, 95%CI 1.40–2.34), and procedures performed in England (OR 1.99, 95%CI 1.22–3.36) ( Table 4 ). When modeled categorically, increasing age was also associated with higher odds of polyp detection, although estimates for older age groups were imprecise due to smaller numbers ( Table 4 ). Increased eANDR was also significantly associated with age (OR 1.02, 95%CI 1.00–1.03), male gender (OR 1.68, 95%CI 1.20–2.37), and BCSP group (OR 1.49, 95%CI 1.05–2.09) ( Table 4 ). Given that symptomatic procedures represented a heterogeneous subgroup, sensitivity analyses restricted to surveillance colonoscopies were performed and demonstrated similar findings to the primary analyses ( **Table 3s** ).

**Table:** **Table 4** Multivariable logistic regression analysis for polyp and advanced neoplasia detection.

Variable	PDR, %	Polyp detection			eANDR, % ^1^	Advanced neoplasia detection ^1^		
		OR ^2^	95%CI	*P* value		OR ^2^	95%CI	*P* value
Age, years (continuous)		1.03	1.03–1.04	<0.001		1.02	1.00–1.03	0.02
Age, years (categorical)								
18–24	16.0	Ref			4.0	Ref		
25–34	27.9	1.49	0.54–5.30	0.48	1.7	0.39	0.06–7.64	0.40
35–44	42.6	2.56	0.94–8.99	0.09	4.5	1.06	0.21–19.33	0.96
45–54	45.9	3.36	1.23–11.76	0.03	4.8	1.16	0.23–21.11	0.89
55–64	52.0	4.24	1.56–14.85	0.01	4.8	1.22	0.24–22.25	0.85
65–74	59.7	6.22	2.26–21.91	0.001	5.4	1.35	0.26–24.78	0.77
75–84	52.8	5.36	1.82–19.76	0.005	4.8	1.27	0.19–25.03	0.83
≥85	75.0	13.9	1.37–330.14	0.04	0.0	0.00	0.00–0.00	0.98
Gender								
Female	44.0	Ref			3.6	Ref		
Male	51.3	1.48	1.26–1.73	<0.001	5.8	1.68	1.20–2.37	0.003
BCSP group								
Non-BCSP	43.3	Ref			5.6	Ref		
BCSP	53.7	1.50	1.28–1.77	<0.001	3.9	1.49	1.05–2.09	0.02
Indication								
Surveillance	47.4	Ref			4.3	Ref		
Symptomatic	38.9	0.73	0.54–0.99	0.047	6.4	1.48	0.80–2.54	0.18
Bowel preparation quality								
Inadequate	36.2	Ref						
Adequate	48.0	1.81	1.40–2.34	<0.001				
Nation								
Scotland	29.7	Ref			3.1			
Northern Ireland	32.1	1.32	0.45–3.62	0.60	4.5			
Wales	33.5	1.27	0.70–2.34	0.44	3.6			
England	48.4	1.99	1.22–3.36	<0.001	5.1			
BCSP, Bowel Cancer Screening Programme; eANDR, endoscopic advanced neoplasia detection rate; OR, odds ratio; PDR, polyp detection rate. ^1^ eANDR is endoscopically defined as polyps ≥10 mm or endoscopically coded colorectal cancer. ^2^ The multivariable logistic regression model for polyp detection was adjusted for age (continuous), gender, BCSP status, indication, bowel preparation quality, and nation, whereas the multivariable logistic regression model for eANDR was adjusted for age (continuous), gender, BCSP status, and indication. Separate models replacing age as a continuous variable with age categories were fitted to estimate age group-specific adjusted ORs using the same covariates.								

## Discussion

This analysis of 3196 colonoscopies across 288 UK endoscopy units provides the first national procedural-level evaluation of colonoscopy quality and endoscopic yield in Lynch syndrome using NED between June 2024 and June 2025. Overall quality standards were high, with a 97.6% completion rate, adequate bowel preparation in 89.0%, and adequate procedure tolerance in 96.3%. Furthermore, median withdrawal time was 13.1 minutes for all procedures and 11.0 minutes for negative colonoscopies, which were longer than typically reported in average-risk cohorts and consistent with expected standards of high-risk surveillance 5 28 . These quality indicators likely contributed to the high colonoscopic yield observed, with a PDR of 46.8% and an eANDR of 4.7%.

Although PDR is not routinely used as a quality metric in Lynch syndrome surveillance, our results are comparable to PDRs (36.0%–44.4%) reported in the linked color imaging/white-light endoscopy trial conducted under high-quality surveillance conditions 29 . Moreover, ADR is a validated key performance indicator and inversely associated with the risk of post-colonoscopy CRC 17 . Using a validated PDR:ADR conversion factor, our estimated ADR (32.2%) aligns with the range from Lynch syndrome cohorts undergoing high-quality surveillance (28%–42%), including the TIMELY trial (ADR 33%–36%) and the Sánchez et al. study, which reported gene-specific ADRs ranging from 39.4% to 41.9% 19 28 30 31 32 . Together, our data reaffirm the impact of high-quality colonoscopic practice and endoscopic yield expected in Lynch syndrome when quality standards are met.

However, high polyp detection may not necessarily translate into proportional reductions in CRC risk in Lynch syndrome. Unlike in average-risk populations, Lynch syndrome-related carcinogenesis may follow one of three molecular pathways: 1) conventional adenoma–carcinoma sequence with later acquisition of MMR deficiency; 2) MMR deficiency occurring at an early stage, resulting in an accelerated adenoma–carcinoma progression; and 3) MMR-deficient submucosal crypts progressing directly to carcinoma without a visible adenomatous precursor 18 . Furthermore, neoplasia detection rates may vary by genotype, with Sánchez et al. reporting higher advanced adenoma detection among *MLH1/MSH2* (14.5%) vs. *MSH6/PMS2* (11.9%) carriers 31 . Consequently, post-colonoscopy CRC may occur despite high-quality surveillance. Nonetheless, adherence to high-quality colonoscopic surveillance remains essential to maximize the detection of precancerous lesions amenable to endoscopic therapy and identify early cancers where timely intervention may reduce morbidity.

We confirmed the well-described pattern of polyp burden in Lynch syndrome whereby flat and right-sided polyps are commonly detected, highlighting the inherent challenges of neoplasia detection in this population 33 . Although most procedures identified 1–2 lesions (mean number of polyps per procedure 1.08, and 2.29 among colonoscopies with polyps), a considerable proportion were diminutive (<5 mm). However, such subtle lesions can harbor high grade dysplasia and be missed without careful mucosal inspection, further reinforcing the need for rigorous surveillance standards in Lynch syndrome 33 34 . Consistent with BSG and ESGE guidelines, we recommend avoiding withdrawal times of <6 minutes and instead adopting a practical minimum target of 10 minutes and considering an aspirational target of 12–15 minutes for Lynch syndrome surveillance 5 6 . Notably, a UK Lynch syndrome surveillance cohort reported a median overall withdrawal time of 15 minutes, while 55.8% of negative procedures had a withdrawal time of ≥10 minutes in our study, supporting the feasibility of achieving these benchmarks in routine practice 28 .

BCSP procedures accounted for 34% of colonoscopies, which likely represents the early phase of Lynch syndrome BCSP implementation in England, with many individuals continuing to undergo surveillance under symptomatic services following established local pathways, and practical considerations including patient convenience and limited awareness. This proportion is likely to increase in the coming years with greater integration of the Lynch syndrome BCSP. Importantly, BCSP colonoscopies consistently outperformed non-BCSP counterparts across multiple quality indicators, including completion rate, bowel preparation quality, and longer withdrawal time 35 . Correspondingly, PDR, eANDR, and mean number of polyps per procedure were significantly higher among BCSP procedures, with an estimated ADR of 36.9% vs. 29.8% among non-BCSP procedures, which may reflect rigorous accreditation and quality-assured processes embedded within the screening program rather than BCSP status in isolation 22 23 .

Multivariable logistic regression demonstrated that increasing age and male gender, BCSP providers, adequate bowel preparation, and procedures performed in England were key factors associated with higher polyp detection in Lynch syndrome. In contrast, only increasing age, male gender, and BCSP groups were significant variables that increased eANDR. These associations may arise from differences in underlying neoplasia prevalence and other factors affecting lesion detection, while inter-nation variation likely represents unmeasured differences in service organization and geographical heterogeneity rather than a causal determinant of PDR. Our data support the incorporation of colonoscopic surveillance within a structured quality-assured screening pathway, which may standardize surveillance quality and promote equitable access to care through established mechanisms for accreditation, monitoring, and recall. However, as Lynch syndrome surveillance in devolved nations remains outside BCSP, there is an opportunity to extend the BCSP infrastructure, governance, and quality assurance to achieve equitable and consistent surveillance outcomes across the UK. More broadly, the UK experience may provide a framework for the development of quality-assured Lynch syndrome surveillance programs internationally.

Patient comfort is critical for maintaining long-term adherence to surveillance. In our cohort, most colonoscopies (96.3%) were well tolerated. Conscious sedation was used in 71.2% of examinations, which was similar to the UK audit figure of 69.1%, while general anesthesia was used in 2.2% of procedures 8 . There were no significant differences in sedation use and comfort levels between BCSP and non-BCSP providers, suggesting that high standards of patient comfort are achieved nationally. However, interpretation of these findings should be tempered by the fact that comfort levels and other quality indicators are reported by endoscopists and may be subject to reporting bias. Future work incorporating patient-reported experience and outcomes would provide valuable insights on tolerability, barriers to attendance, and opportunities to optimize patient-centered care.

This study has several limitations. First, the absence of patient-level identifiers precluded the verification of repeated procedures performed within the study period, although the 1-year extraction window may have helped to minimize this effect. Furthermore, NED does not capture interval history, genetic variant, previous CRC, or colorectal resection information beyond procedural extent and has no linkage with histopathology, limiting our ability to evaluate interval cancers, and gene-specific and post-surgical diagnostic outcomes. Without linked histopathology and staging information, CRC-specific outcomes derived from procedure-level data are difficult to interpret. Given that CRC in Lynch syndrome is frequently diagnosed at an early stage and may present endoscopically as malignant or advanced polyps, this may result in misclassification of polyp cancers and underreporting of CRC detection rates. CRCs may often occur prior to establishing a Lynch syndrome diagnosis, further underestimating the cancer prevalence in this group. For these reasons, we focused on reporting advanced neoplasia detection as a more reliable composite outcome measure instead of CRC detection rate.

Although NED provides standardized real-time data capture on procedure-level information, it is reliant on accurate and complete documentation. Non-mandatory fields such as use of chromoendoscopy, artificial intelligence, and endoscopic adjuncts may be prone to missing or inconsistent entries and were not reported. Furthermore, misclassification of BCSP or Lynch syndrome procedures may occur when the pathway is not explicitly coded. Finally, NED coverage is still expanding and the dataset does not include all UK providers, particularly within devolved nations and with limited coverage in Northern Ireland; this may introduce selection bias. However, lower contributions of procedures from devolved nations is consistent with previously reported differences in NED uploads and regional differences should be interpreted in the context of variation in NED coverage and service configuration across the UK 13 . Furthermore, as BCSP status was defined according to procedure-level coding and non-BCSP procedures may also be undertaken in BCSP-accredited units, the potential for center-level selection bias cannot be excluded. We were nonetheless able to report findings from 54.9% of all UK providers, which may in part reflect the delivery of Lynch syndrome surveillance within specialist/high-volume centers and incomplete data capture from independent/private sector providers.

This national dataset constitutes the largest and near-complete appraisal of Lynch syndrome colonoscopy quality and endoscopic yield in the UK, providing important evidence to support benchmarking of Lynch syndrome-specific quality standards. Future analyses leveraging longitudinal outcomes from Lynch syndrome BCSP, integrating data on gene variants, patient-level factors, and histopathology amalgamation, will be essential in further refining colonoscopy performance metrics and generating robust evidence to support genotype-tailored surveillance.

In conclusion, our study demonstrates a high overall colonoscopy performance across the UK and superior quality and endoscopic yield among BCSP procedures in Lynch syndrome. These findings lay the groundwork for developing Lynch syndrome-specific quality benchmarks and potential for colonoscopic surveillance under the Lynch syndrome BCSP to support the delivery of high-quality care for this population.

## Declaration of GenAI Use

The authors used ChatGPT (OpenAI) solely to assist with troubleshooting R coding errors and proofreading of the manuscript, in accordance with Thieme’s guidance on critical and responsible use of Generative AI. All analyses, interpretation of results, study design, and content were independently undertaken by the authors, who take full responsibility for the final manuscript.

## Supplementary Material

Supplementary Material Zusatzmaterial

## References

[1] Win AK Jenkins MA Dowty JG Prevalence and penetrance of major genes and polygenes for colorectal cancer Cancer Epidemiol Biomarkers Prev 2017 26 404 412 10.1158/1055-9965.EPI-16-0693 27799157 PMC5336409

[2] Grzymski JJ Elhanan G Morales Rosado JA Population genetic screening efficiently identifies carriers of autosomal dominant diseases Nat Med 2020 26 1235 1239 10.1038/s41591-020-0982-5 32719484

[3] Järvinen HJ Aarnio M Mustonen H Controlled 15-year trial on screening for colorectal cancer in families with hereditary nonpolyposis colorectal cancer Gastroenterology 2000 118 829 834 10.1016/s0016-5085(00)70168-5 10784581

[4] Monahan KJ Bradshaw N Dolwani S Guidelines for the management of hereditary colorectal cancer from the British Society of Gastroenterology (BSG)/Association of Coloproctology of Great Britain and Ireland (ACPGBI)/United Kingdom Cancer genetics group (UKCGG) Gut 2020 69 411 444 10.1136/gutjnl-2019-319915 PMC7034349 31780574

[5] Rees CJ Thomas Gibson S Rutter MD UK key performance indicators and quality assurance standards for colonoscopy Gut 2016 65 1923 1929 10.1136/gutjnl-2016-312044 PMC5136732 27531829

[6] Kaminski MF Regula J Kraszewska E Quality indicators for colonoscopy and the risk of interval cancer N Engl J Med 2010 362 1795 1803 10.1056/NEJMoa0907667 20463339

[7] Kaminski MF Thomas-Gibson S Bugajski M Performance measures for lower gastrointestinal endoscopy: a European Society of Gastrointestinal Endoscopy (ESGE) Quality Improvement Initiative Endoscopy 2017 49 378 397 10.1177/2050640617700014 28268235

[8] Beaton D Sharp L Trudgill N British Society of Gastroenterology national evaluation of colonoscopy quality: findings from the National Endoscopy Database Gastrointest Endosc 2025 102 117 1.28E8 10.1016/j.gie.2024.12.038 39788213

[9] Lee TJW Siau K Esmaily S Development of a national automated endoscopy database: The United Kingdom National Endoscopy Database (NED) United European Gastroenterol J 2019 7 798 806 10.1177/2050640619841539 31316784 PMC6620868

[10] Catlow J Sharp L Wagnild J Nationally automated colonoscopy performance feedback increases polyp detection: The NED APRIQOT randomized controlled trial Clin Gastroenterol Hepatol 2024 22 1926 1936 10.1016/j.cgh.2024.03.048 38759827

[11] Beaton DR Sharp L Lu L Diagnostic yield from symptomatic gastroscopy in the UK: British Society of Gastroenterology analysis using data from the National Endoscopy Database Gut 2024 73 1421 1430 10.1136/gutjnl-2024-332071 38697772

[12] Beaton D Sharp L Lu L Diagnostic yield from symptomatic lower gastrointestinal endoscopy in the UK: a British Society of Gastroenterology analysis using data from the National Endoscopy Database Aliment Pharmacol Ther 2024 59 1589 1603 10.1111/apt.18003 38634291

[13] Beaton D Sharp L Trudgill NJ UK endoscopy workload and workforce patterns: is there potential to increase capacity? A BSG analysis of the National Endoscopy Database Frontline Gastroenterol 2023 14 103 110 10.1136/flgastro-2022-102145 PMC9933584 36818791

[14] Schwarz S Hornschuch M Pox C Polyp detection rate and cumulative incidence of post-colonoscopy colorectal cancer in Germany Int J Cancer 2023 152 1547 1555 10.1002/ijc.34375 36444495

[15] Francis DL Rodriguez-Correa DT Buchner A Application of a conversion factor to estimate the adenoma detection rate from the polyp detection rate Gastrointest Endosc 2011 73 493 497 10.1016/j.gie.2011.01.005 21353846

[16] Niv Y Polyp detection rate may predict adenoma detection rate: a meta-Analysis Eur J Gastroenterol Hepatol 2018 30 247 251 10.1097/MEG.0000000000001062 29293111

[17] Corley DA Jensen CD Marks AR Adenoma detection rate and risk of colorectal cancer and death N Engl J Med 2014 370 1298 1306 10.1056/NEJMoa1309086 24693890 PMC4036494

[18] Ahadova A Gallon R Gebert J Three molecular pathways model colorectal carcinogenesis in L ynch syndrome Int J Cancer 2018 143 139 150 10.1002/ijc.31300 29424427

[19] Sánchez A Roos VH Navarro M Quality of colonoscopy is associated with adenoma detection and postcolonoscopy colorectal cancer prevention in Lynch syndrome Clin Gastroenterol Hepatol 2022 20 611 6.21E11 10.1016/j.cgh.2020.11.002 33157315

[20] Monahan KJ Fleming P Ryan NAJ Outcomes from the English National Lynch Syndrome transformation project Int J Cancer 2026 158 2369 2379 10.1002/ijc.70330 PMC12963710 41521736

[21] Monahan KJ Poo SX Lalloo F Improving care for Lynch syndrome patients: integrating surveillance into England’s national Bowel Cancer Screening Programme Fam Cancer 2026 25 25 10.1007/s10689-026-00539-2 41746524 PMC12946326

[22] Logan RFA Patnick J Nickerson C Outcomes of the Bowel Cancer Screening Programme (BCSP) in England after the first 1 million tests Gut 2012 61 1439 1446 10.1136/gutjnl-2011-300843 22156981 PMC3437782

[23] Joint Advisory Group for GI Endoscopy (JAG) Bowel cancer screener accreditation https://www.bcsa.thejag.org.uk/

[24] Kuipers RN Burggraaff MF Maas MHJ Endoscopic surveillance for colorectal cancer and its precursor lesions in Lynch syndrome; time for some policy shifts? Hered Cancer Clin Pract 2025 23 13 10.1186/s13053-025-00312-z 40241188 PMC12001557

[25] Huntley C Loong L Mallinson C Impact of surveillance colonoscopy on colorectal cancer incidence and mortality in Lynch syndrome: a national observational cohort study of patients in the English NHS 2010–2022 Gut 2026 10.1136/gutjnl-2025-337379 42562418

[26] Huntley C Loong L Mallinson C The comprehensive English National Lynch Syndrome Registry: development and description of a new genomics data resource EClinicalMedicine 2024 69 102465 10.1016/j.eclinm.2024.102465 38356732 PMC10864212

[27] Chino A Tanakaya K Nakajima T Colorectal cancer and advanced adenoma characteristics according to causative mismatch repair gene variant in Japanese colorectal surveillance for Lynch syndrome J Gastroenterol 2024 59 699 708 10.1007/s00535-024-02128-5 38902413

[28] Poo SXW Cuthill V Walton J The diagnostic yield of colonoscopy surveillance in Lynch syndrome Frontline Gastroenterol 2025 10.1136/flgastro-2025-103281

[29] Houwen BBSL Hazewinkel Y Pellisé M Linked colour imaging for the detection of polyps in patients with Lynch syndrome: a multicentre, parallel randomised controlled trial Gut 2022 71 553 560 10.1136/gutjnl-2020-323132 PMC8862075 34086597

[30] Rivero-Sánchez L Arnau-Collell C Herrero J White-light endoscopy is adequate for Lynch syndrome surveillance in a randomized and noninferiority study Gastroenterology 2020 158 895 9040 10.1053/j.gastro.2019.09.003 31520613

[31] Sánchez A Castillo-Iturra J Roos VH Gene-specific detection rate of adenomas and advanced adenomas in Lynch syndrome Gastroenterology 2025 169 663 675 10.1053/j.gastro.2025.03.042 40315961

[32] Ortiz O Daca-Alvarez M Rivero-Sanchez L An artificial intelligence-assisted system versus white light endoscopy alone for adenoma detection in individuals with Lynch syndrome (TIMELY): an international, multicentre, randomised controlled trial Lancet Gastroenterol Hepatol 2024 9 802 810 10.1016/S2468-1253(24)00187-0 39033774

[33] Lindgren G Liljegren A Jaramillo E Adenoma prevalence and cancer risk in familial non-polyposis colorectal cancer Gut 2002 50 228 234 10.1136/gut.50.2.228 11788565 PMC1773092

[34] De Jong AE Morreau H Van Puijenbroek M The role of mismatch repair gene defects in the development of adenomas in patients with HNPCC Gastroenterology 2004 126 42 48 10.1053/j.gastro.2003.10.043 14699485

[35] Lee TJW Rees CJ Blanks RG Colonoscopic factors associated with adenoma detection in a national colorectal cancer screening program Endoscopy 2014 46 203 211 10.1055/s-0033-1358831 24473907

