# Supplementary material for: Benchmarking UK Lynch syndrome colonoscopy surveillance: quality metrics and diagnostic yield from the National Endoscopy Database
Source: Endoscopy. 2026 Aug 31;58(11):1223–34. doi: 10.1055/a-2939-3426 (PMC13758998; doi:10.1055/a-2939-3426)
Supplement: Supplementary file 1 [file 10-1055-a-2939-3426_29436866.pdf]

Supplementary material

Benchmarking UK Lynch syndrome colonoscopy surveillance: quality metrics and diagnostic yield from the National Endoscopy Database

Stephanie X. W. Poo, Thomas Lee, Amanda J. Cross, Kevin J. Monahan

Table 1s Table of definitions according to the British Society of Gastroenterology quality standards[1].

Complete procedures	A complete procedure is defined as a colonoscope successfully reaching the intended extent of the bowel, including the caecum or terminal ileum for patients with intact colons and ileo-colic anastomosis or neo-terminal ileum intubation in cases where the caecum has been surgically removed.
Bowel preparation	Bowel preparation of each colonoscopy is classified according to the Boston Bowel Preparation Score (BBPS). Excellent bowel preparation is defined according to a BBPS of 8 or more, while Good bowel preparation is defined as having a BBPS of 6 or 7. Adequate bowel preparation is defined as those with Excellent or Good bowel preparation, with an overall BBPS score of 6 and above.
Procedure tolerance	Adequate tolerance is defined as encounters where there was no, minimal or mild discomfort during procedures.
Withdrawal time	Withdrawal time is the duration of time taken to withdraw the colonoscope from the furthest point of insertion to scope removal. Negative colonoscopy (NC)-withdrawal time is the time taken to extubate the colonoscope from the furthest point of insertion to scope removal in procedures without polypectomy or therapeutic intervention.
Conscious sedation	Conscious sedation is defined as the use of intravenous sedative agents, typically midazolam and/or fentanyl to achieve patient comfort while maintaining verbal responsiveness and spontaneous ventilation
Polyp detection rate	The number of colonoscopies which has at least one polyp detected out of all the colonoscopies performed
Endoscopically-defined advanced neoplasia detection rate (eANDR)	The proportion of colonoscopies which detected polyps of at least 10mm in size and/or colorectal cancer out of all the colonoscopies performed
Polyp morphology	Morphology was classified according to Paris classification, with Paris Is polyps being sessile, II and lateral spreading lesions being flat, and 1sp and 1p being classified as pedunculated polyps
Polyp location	Right colonic polyps indicate polyps detected proximal to the splenic flexure, whereas left colonic polyps are polyps found from the recto-sigmoid junction to the splenic flexure. Rectal polyps are polyps identified in the rectum.

[1] Rees CJ, Thomas Gibson S, Rutter MD et al. UK key performance indicators and quality assurance standards for colonoscopy. Gut 2016; 65: 1923–1929. doi:10.1136/GUTJNL-2016-312044

Table 2s Characteristics of endoscopists completing Lynch syndrome colonoscopies to the National Endoscopy Database during the study period, stratified by BCSP status.

**Number of endoscopists**	**Total N=978**	**≥1 BCSP N=239**	**No BCSP N=739**	**P-value**
**No. of procedures during study period, median (IQR)**	348 (212-548)	415 (284-614)	315 (183-526)	<0.001
**Procedure volume by category, n (%)**				<0.001
<100	66 (6.7)	2 (0.8)	64 (8.7)	
100-199	160 (16.4)	15 (6.3)	145 (19.6)	
200-299	182 (18.6)	48 (20.1)	134 (18.1)	
300-399	154 (15.7)	45 (18.8)	109 (14.7)	
400-499	132 (13.5)	46 (19.2)	86 (11.6)	
500-599	75 (7.7)	20 (8.4)	55 (7.4)	
≥600	209 (21.4)	63 (26.4)	146 (19.8)	
**Endoscopist group**				<0.001
Consultant	672	182	490	
Median procedures (IQR)	316 (203-472)	373.5 (265-484)	297.5 (176-468)	
Clinical Endoscopist	214	48	166	
Median procedures (IQR)	526 (337-763)	706.5 (535-832)	466.5 (313-694)	
Non-consultant/Other staff	51	7	44	
Median procedures (IQR)	319 (166-659)	697 (393-828)	290 (162-596)	
Trainee	40	1	39	
Median procedures (IQR)	134 (78.2-230)	435 (435-435)	134 (76-210)	
Unknown	1	1	0	

Abbreviations: BCSP, Bowel Cancer Screening Programme; IQR, Interquartile range

≥1 BCSP denotes endoscopists performing ≥1 BCSP-labelled Lynch syndrome procedure during the study period, while No BCSP indicates endoscopists performing no BCSP procedures during the study period.

Endoscopy | DOI 10.1055/a-2939-3426 | © The Author(s).

Table 3s Sensitivity analysis restricted to surveillance colonoscopies: multivariable logistic regression model for factors associated with polyp and advanced neoplasia detection.

Variable	Polyp detection			Advanced neoplasia detection[+]		
	OR*	95%CI	p-value	OR*	95%CI	p-value
**Age, years (continuous)**	1.03	1.03-1.04	<0.001	1.01	1.00-1.03	0.034
**Gender**						
**Female**	Ref			Ref		
**Male**	1.45	1.23-1.70	<0.001	1.60	1.12-2.29	0.010
**BCSP group**						
**Non-BCSP group**	Ref			Ref		
**BCSP group**	1.49	1.26-1.77	<0.001	1.58	1.10-2.27	0.013
**Bowel preparation quality**						
**Inadequate**	Ref					
**Adequate**	1.82	1.39-2.38	<0.001			
**Nation**						
**Scotland**	Ref					
**Northern Ireland**	1.48	0.50-4.16	0.465			
**Wales**	1.36	0.74-2.55	0.329			
**England**	2.13	1.28-3.66	<0.001			

Abbreviations: BCSP, Bowel Cancer Screening Programme; CI, confidence interval; OR, odds ratio

*The multivariable logistic regression model for polyp detection was adjusted for age (continuous), gender, BCSP status, bowel preparation quality and nation, whereas the multivariable logistic regression model for advanced neoplasia detection was adjusted for age (continuous), gender, and BCSP status.
[+]Advanced neoplasia detection rate is endoscopically defined as polyps ≥10mm in size or endoscopically coded colorectal cancer
